# Uniform wet-Spinning Mechanically Automated (USMA) fiber device

**DOI:** 10.1016/j.ohx.2020.e00124

**Published:** 2020-07-23

**Authors:** Alexander N. Mitropoulos, Kylor T. Kiesewetter, Eric Horne, Jeff Butler, Joseph R. Loverde, J. Kenneth Wickiser

**Affiliations:** a Department of Mathematical Sciences, United States Military Academy, West Point, NY 10996, USA; b Department of Chemistry and Life Science, United States Military Academy, West Point, NY 10996, USA; c Department of Civil and Mechanical Engineering, United States Military Academy, West Point, NY 10996, USA; d Academic Research Division, United States Military Academy, West Point, NY 10996, USA

**Keywords:** Wet spinning, Biomaterials, Biopolymers, Collagen, Biotextile, Fiber

## Abstract

Bioengineering techniques for producing fibers from biomaterials is a growing requirement in medical device technology research and development environments. Scale-up and control of diameter, shape, and length of fibrous proteins and elastomeric polymers are essential to produce defined and consistent materials for experimentation and clinical use. Here, we developed a novel wet spinning fiber extruder and spooler system engineered to draw precipitated fibers several meters in length across five spools. By controlling both the extrusion and spooling rate, the diameter of the fiber can be controlled on the order of 10–1000 µm. Using this system, we extruded and spooled precipitated Type-1 Collagen fibers up to 7.5 m in length on a single spool with a controllable diameter range of 30–50 µm. Furthermore, this device facilitated bundling of fibers directly on the spool in order to create 1–12 cm long fiber bundles for experimentation. This system may be used in the laboratory to scale up biomaterial fiber production to produce degradable scaffolds made from synthetic or natural materials for a range of biomedical applications.

## Graphical abstract

**Figure:**
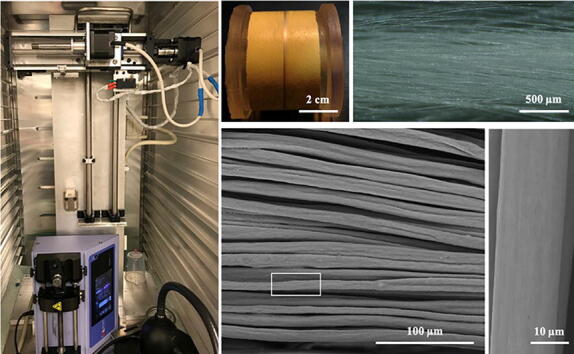

Specifications table

**Table:** 

Hardware name	*Uniform wet-Spinning Mechanically Automated (USMA) Fiber Device*
Subject area	• Engineering and Material Science • Chemistry and Biochemistry • Medical Devices • Neuroscience • Biological Sciences (e.g. Microbiology and Biochemistry) • General
Hardware type	• Biological sample handling and preparation • Mechanical engineering and materials science
Open Source License	*CC0 1.0 Universal*
Cost of Hardware	*$2,100.00*
Source File Repository	https://doi.org/10.17605/OSF.IO/RKY2S

## 1 Hardware in context

Biomaterial fibers are increasingly relied upon in tissue engineering projects to mimic tissue structures given that degradable and biocompatible polymers are derived from natural materials. In normal biochemical processes, proteins are extracted and purified in aqueous form, but for medical device and implantable studies, the polymers need to be returned to a working solid to benefit tissue engineering applications. Many tissue-like structures currently exist in tissue engineering applications such as hydrogels, foams, and films [1], [2], [3]. These forms have been well-studied and are easy to fabricate in bulk quantities using standard chemistry and gelation-casting techniques [3], [4], [5]. However, it is currently a challenge to develop three-dimensional structures with large aspect ratios such as individual fibers with micrometer-scale diameters that mimic the natural tissue made from protein and biopolymer materials; these large aspect ratio fibers offer a unique directional approach to culturing and guiding the growth, development, and locomotion of cells. One such method to produce continuous solid fibers with the dimensions of natural tissue is electrospinning, which uses voltage separation to extrude fibers into mats, producing fibers from 100 nm to several microns in diameter [6], [7], [8], [9]. Alignment of electrospinning fibers has been attempted recently with high levels of success made from several degradable materials, but still only produces a mat that is uncontrolled and cannot be separated into individual fibers [6], [7]. Additionally, electrospinning requires specific molecular length polymers with the necessary viscosity and material properties to extrude a continuous fiber onto a charged surface which, during formation, can change and damage the innate molecular structure of the nascent fibers [7]. Therefore, other methods that produce fibers in liquid environments are needed to generate fibers with larger diameters that can be used for other applications as solid physical features to determine factors such as cell fate and differentiation [10], [11], [12].

Collagen is one of the most common materials used as an engineered scaffold as it is integral to mammalian extracellular matrix (ECM) and offers the required chemical and mechanical properties to anchor multiple cell types to a variety of materials [2], [13], [14], [15]. The standard preparation method to generate collagen fibers from an aqueous solution uses a wet spinning technique by chemically crosslinking the collagen allowing it to maintain its mechanical properties and be used as a tissue scaffold [10], [12]. ECM proteins are normally fibrillar in nature and material engineers of such scaffolds try to mimic their natural structure [12]. Fibers have the unique capability in that they can be used as biotextiles and be used in knitting, braiding, and weaving [12]. However, these fibers are not available for purchase, especially for researchers who want to modify the materials (such as the surface or bulk) for more specific tissue engineering applications. Therefore, it would benefit the tissue engineering community to develop an inexpensive, simple, and reliable device that both provides a tunable wet spinning process and allows for customization based on a project’s requirements.

Here we have designed and built a wet spinning fiber device that is modular that can control fiber type and diameter by changing the wet spinning bath, the extrusion material, and the extrusion rate to capture the as-made fibers. Herein we present the technical details and the rationale behind engineering this hardware and software control system [12], [16]. We show the specific hardware development of an automated and controlled wet spinning fiber device to form collagen fibers 5 to 30 µm in diameter spanning several meters in length. We highlight the flexibility designed into the system to allow for the production of other natural and synthetic fibers with significantly different properties.

## 2 Hardware description

The device was designed in order to produce highly aligned and continuous fibers with controlled diameter and length by controlling the rate of extrusion, the time in the wet spinning bath, and the drying time (the time it takes for the fiber to move from the extrusion bath to the spool). The device is also able to assist with fiber bundling by controlling the fiber length spooled on each spool (i.e. 10 rotations of fiber per spool). The fiber engineering system is composed of two major components, including the material extruder and the spooler which are held in alignment.

For extrusion, we used a commercially available Harvard Apparatus II Elite Syringe Pump, which can be interchanged with any syringe pump or extrusion mechanism that allows extrusion of aqueous material. This pump works in parallel with control of the spool drawing rate set by the spooling stepper motor. We arranged the syringe pump in the vertical direction, which allowed us to mount a customized extrusion needle / bath assembly directly to the syringe. In this arrangement, aqueous fiber is extruded within a bath and then mechanically manipulated drawn by the spooling mechanism. The user-defined vertical space between the extruder and the spooler allows application of natural shearing force to the fiber as it dries prior to spooling.

For spooling, we built hardware consisting of two SureStep stepper motors controlled by an Arduino UNO. The stepper motors are arranged such that one performs spooling (drawing of the fiber) and the other selects the spool to be drawn (1 of 5 spools). The Arduino is programmed with the spooling rate (to adjust fiber diameter and drying time) and required fiber length to be collected on each spool (number of rotations of fiber per spool).

Researchers who use the USMA Fiber Device system will do so because it offers:

• Automation of fiber making to allow consistency and control of fiber making
• Capability to use varying crosslinking solutions and material to make required fibers
• Easy variation in speed and control of fiber pulling and drying required for fiber formation

## 3 Design files

Design Files Summary

**Table:** 

Design file name	File type	Open source license	Location of the file
Fig. 1	Figure		Available with article
Fig. 2	Figure		Available with article
Fig. 3	Figure		Available with article
Fig. 4	Figure	*CC0 1.0 Universal*	Available with article, https://doi.org/10.17605/OSF.IO/RKY2S
Fig. 5	Figure		Available with article
Spool 01	CAD File	*CC0 1.0 Universal*	https://doi.org/10.17605/OSF.IO/RKY2S
Fiber Spinning software 01	Arduino File	*CC0 1.0 Universal*	https://doi.org/10.17605/OSF.IO/RKY2S

**Fig. 1:**
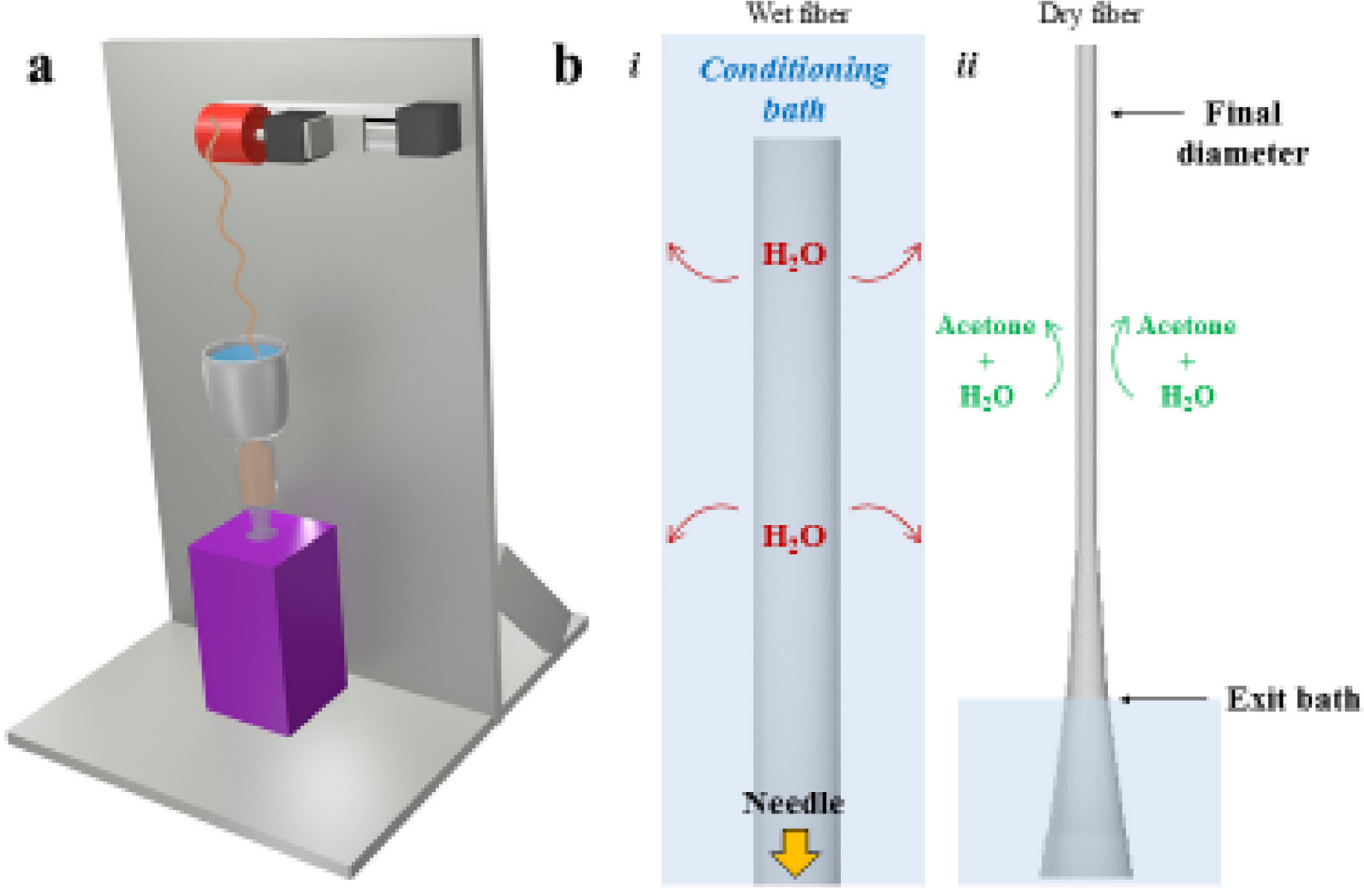
Schematic of fiber making. a) Rendering of USMA fiber device. b) Schematic representation of individual fiber process.

Fig. 1*: Schematic assembly of USMA Fiber Device.*

**Fig. 2:**
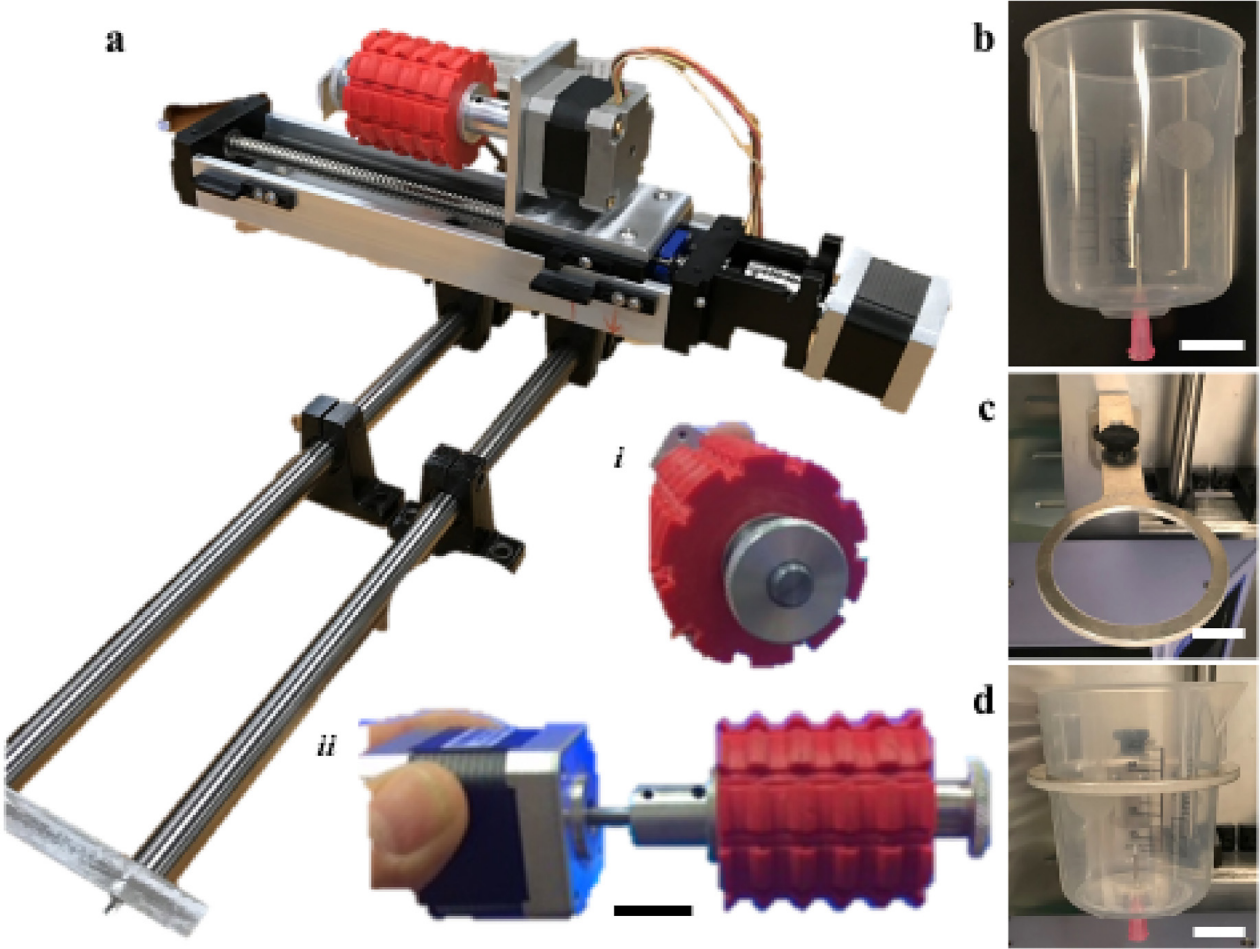
Components of mechanical device. a) Mechanical apparatus of design build showing the stepper motors and linear actuator assembly with (i) spool and (ii) spool attached to the drawing stepper motor. b) Bath cup and needle assembly. c) Holding ring for bath and needle component. d) Bath assembly held by ring assembly.

Fig. 2 *: Images of individual components including the motorized spooling mechanism with spooling dowels, and the bath assembly with needle.*

**Fig. 3:**
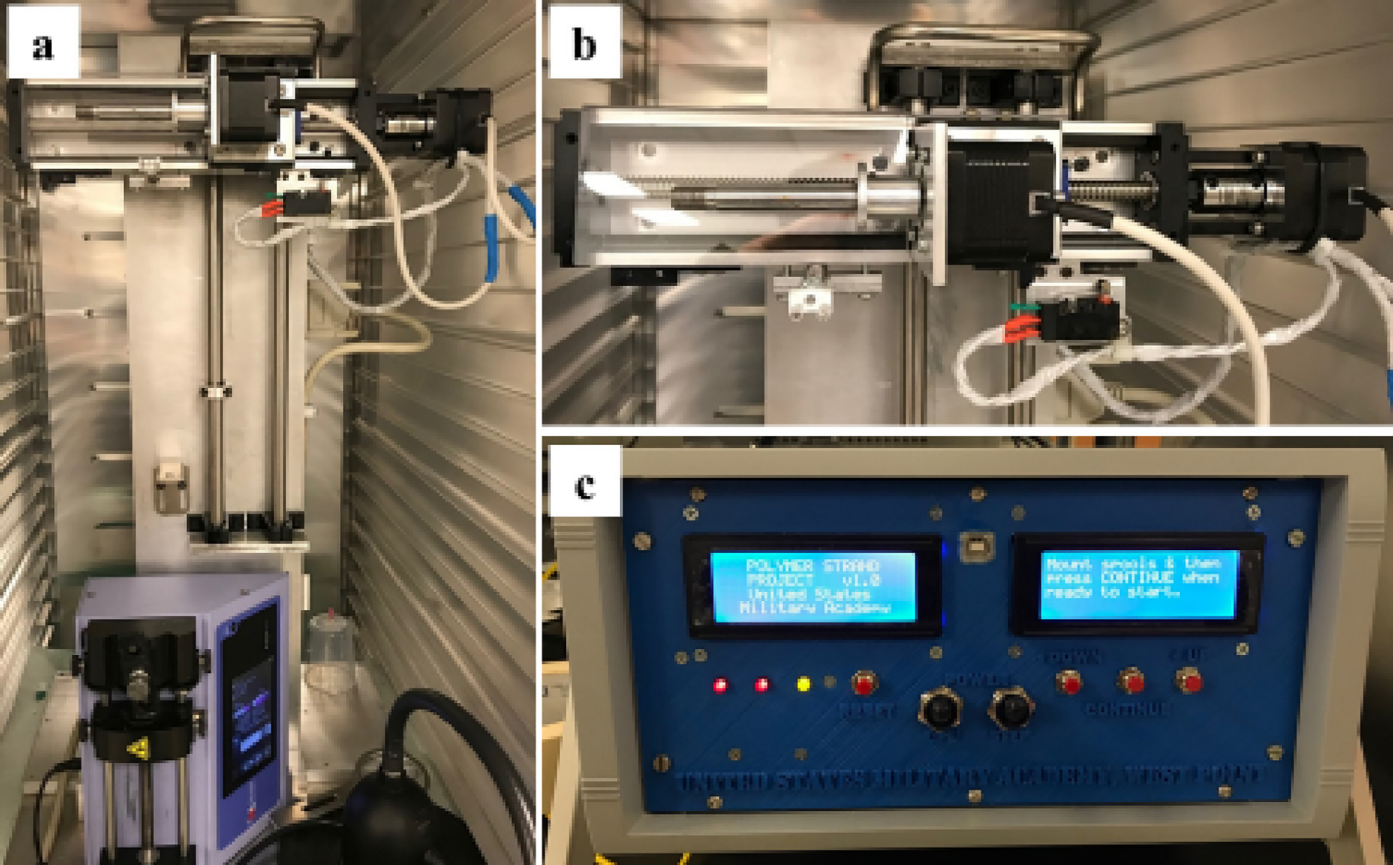
USMA Fiber Device. a) Spooling mechanism in humidity and temperature controlled chamber with syringe pump. b) Close up view of completed spooling assembly with wiring connected to the c) OKW electrical enclosure.

Fig. 3 *: Image of USMA Fiber Device, mechanical controls, and electronic controls.*

**Fig. 4:**
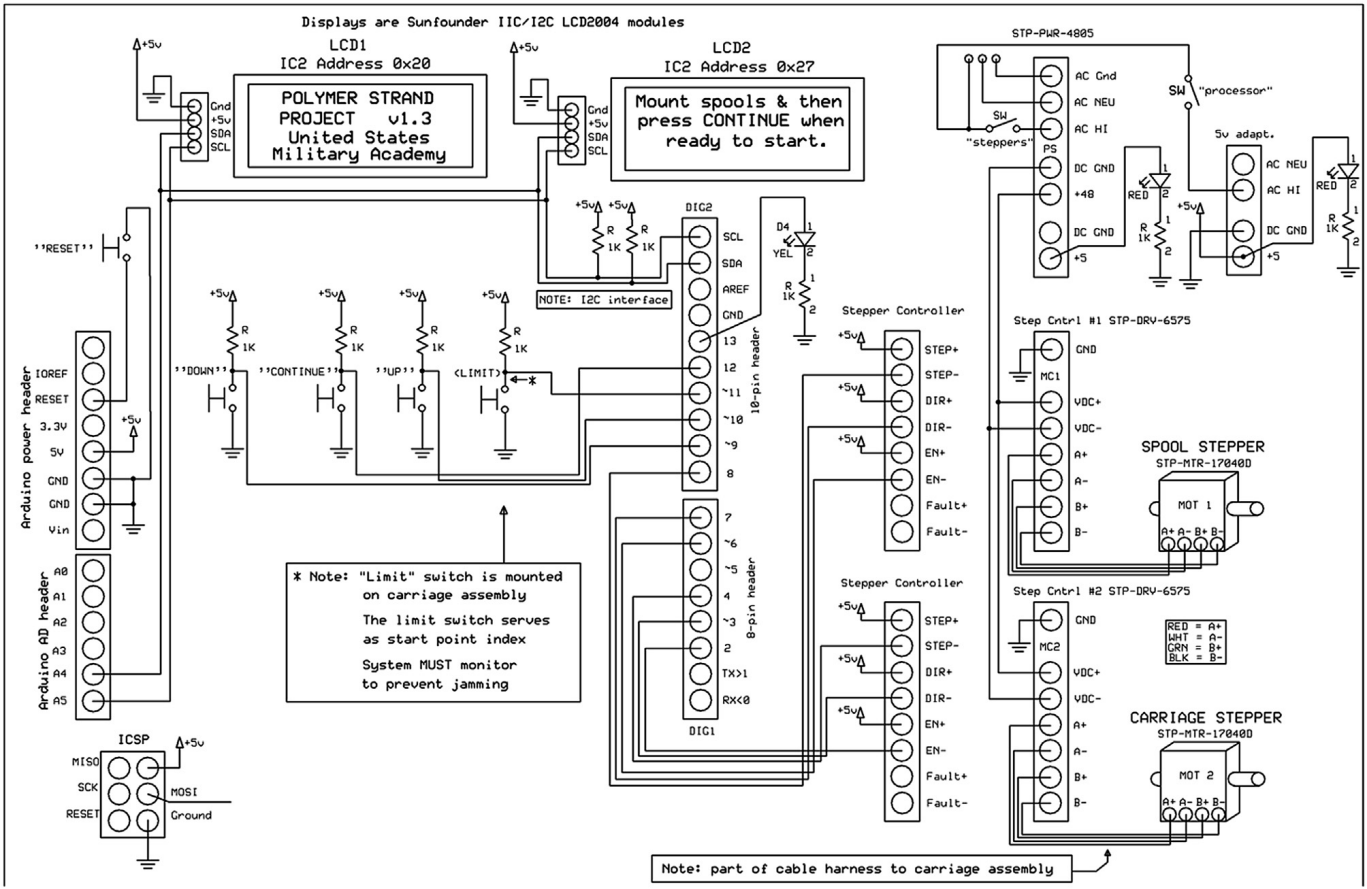
Schematic diagram of electronic connections (https://doi.org/10.17605/OSF.IO/RKY2S).

Fig. 4 *: Schematic diagram of electronic connections.*

**Fig. 5:**
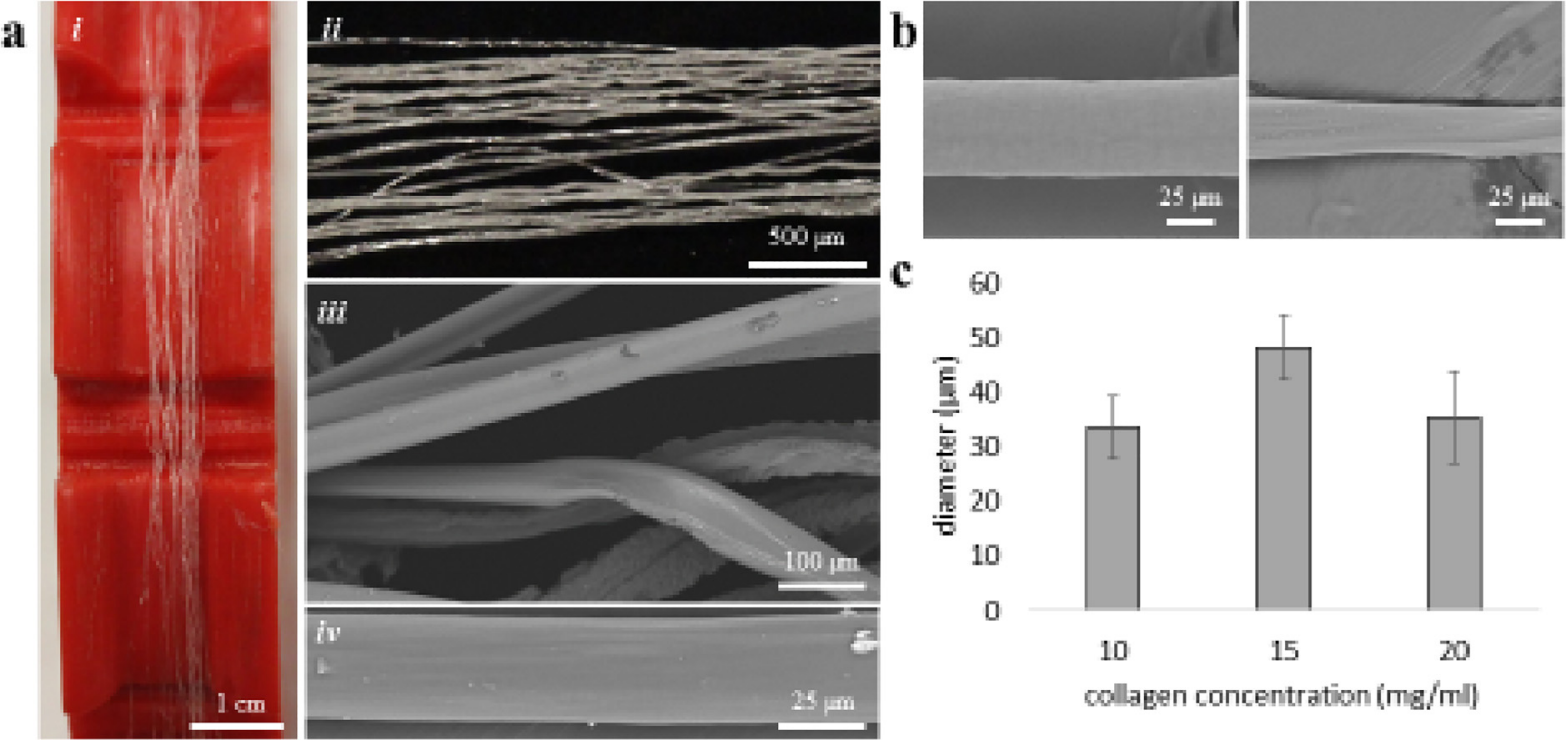
Validation of USMA Fiber Device. a) Fiber alignment after spooling and bundling. (i) Fibers on an individual spool revealing tight alignment and bundling capability on a spool with no alignment control. (ii) Close up view of fiber alignment after bundling. (iii) Scanning electron microscope (SEM) image of aligned fibers with a collagen concentrations of 10 mg/ml with (iv) close up image of a single fiber. b) Images are collagen concentrations of 15 mg/ml (left) and 20 mg/ml (right). c) Diameter control of the USMA Fiber Device for varying concentrations of collagen slurry.

Fig. 5 *: Fiber analysis made from the USMA Fiber Device controlling concentration and needle size.*

Spool 01: Engineered spool allowing for easy wrapping and bundling capabilities.

Fiber Spinning Software 01: Coded Arduino file used to run spooling mechanism. Easily modifiable program to change spooling rate and number of rotations.

## 4 Bill of materials

Bill of Materials

**Table:** 

Designator	Component	Number	Cost per unit -currency	Total cost -currency	Source of materials	Material type
Fiber motor spool	SureMotion linear actuator assembly, value slide, 6in travel, lead screw, 0.2in pitch, hard coated aluminum rail.	1	$789.00 USD	$789.00 USD	Automation Direct (LAVL-60T06LP2)	Non-specific
Fiber motor	SureStep stepper motor, NEMA size 17 frame, IP40, dual shaft, 1.7A, 61 oz-in holding torque, 1.8-degree step angle, 200 steps per revolution, bipolar. (1) 12in cable with 4-lead connector included.	2	$22.00 USD	$44.00 USD	Automation Direct (STP-MTR-17040D)	Non-specific
Fiber motor power supply	SureStep linear power supply, dual output, 48 VDC at 5A unregulated, 5 VDC at 0.5A regulated output, 5A, 240 W, 120/240 VAC, switch selectable, 1-phase, anodized aluminum housing, open frame, wall mount, screw terminals, indoor use only.	2	$140.00 USD	$280.00 USD	Automation Direct (STP-PWR-4805)	Non-specific
Fiber motor driver	SureStep DC microstepping drive, 7.5A per phase, 2-phase, 24–65 VDC, bipolar, MOSFET, dual H-bridge and 4-quadrant output, 200 to 20,000 steps per revolution.	2	$140.00 USD	$280.00 USD	Automation Direct (STP-DRV-6575)	Non-specific
Fiber motor cables	SureStep extension cable, mating connector, 20ft cable length. For use with SureStep motors less than 5A.	2	$15.00 USD	$30.00 USD	Automation Direct (STP-EXT-020)	Non-specific
Device build	Alloy Steel Shoulder Screw 3/16″ Diameter 1″ Long Shoulder, 8–32 Thread	1	$2.22 USD	$2.22 USD	McMaster-Carr (91259A168)	*Metal*
Device build	Highly Corrosion-Resistant 316 Stainless Steel Rod with Certification, 1/4″ Diameter (length 3 ft)	1	$18.16 USD	$18.16 USD	McMaster-Carr (1305T18)	*Metal*
Device build	Clamping Two-Piece Shaft Collar for 1/2″ Diameter, 2024 Aluminum	1	$5.15 USD	$5.15 USD	McMaster-Carr (6436K71)	*Metal*
Device build	Highly Corrosion-Resistant 316 Stainless Steel Strip 0.09″ Thick 1″ × 24″	1	$14.86 USD	$14.86 USD	McMaster-Carr (9090K8)	*Metal*
Device build	Clear Polycarbonate Sheet, 12″ × 12″ × 1/16″	1	$5.32 USD	$5.32 USD	McMaster-Carr (8574K24)	*Polymer*
Electrical wire Support	Fiberwrap Heat-Shrink Tubing Assortments Black	1	$35.16 USD	$35.16 USD	McMaster-Carr (7496K21)	*Polymer*
Device build	Base-Mounted Shaft Support for 1/2″ Shaft Diameter, Iron	8	$27.89 USD	$223.12 USD	McMaster-Carr (6068K23)	*Metal*
Device build	566 Carbon Steel Linear Motion Shaft 1/2″ Diameter, 16″ Long	2	$9.63 USD	$19.26 USD	McMaster-Carr (6061K432)	*Metal*
Device build	Hinged Shaft Collar for 1/2″ Diameter, 303 Stainless Steel	1	$46.97 USD	$46.97 USD	McMaster-Carr (57145K52)	*Metal*
Device build	Sleeve Bearing Block for Washdown Environment 1″ Overall Length UHMW Bearing, for 1/2″ Shaft Diameter	4	$30.00 USD	$120.00 USD	McMaster-Carr (4178K12)	*Metal*
Ceramic guide	Ceramic guide shaft	14	$2.50 USD	$35.00 USD	MB Industries (PG-180LH)	*Ceramic*
Electronic case	OKW Enclosures M6010315-AL Rack; Cabinet; Desktop; 10.5In, 3U, 13.779InDepth; Gray; Aluminum; MetcaseSeries	1	$195.54 USD	$195.54 USD	Electronic Enclosures (**Mfr. Part#:** M6010315-AL, **Allied Stock#:** 70016763)	*Non-specific*
Electronic connectors	Media Bridge USB 2.0 –A Male to B Male cable (6 feet)-High Speed with gold plated connectors-black	1	$5.49 USD	$5.49 USD	Amazon (30-001-06B)	*Non-specific*
Electronic power	ZJchao 9 V 1 A Power Adapter for Arduino (2 flat-pin plug/100CM cable)	1	$5.99 USD	$5.99 USD	Amazon	*Non-specific*
Electronic protector	Sunfounder IIC I2C TWI Serial 2004 20x4 LCD Module Shield for Arduino Uno Mega2560	1	$11.99 USD	$11.99 USD	Amazon	Non-specific
Electronic connectors	Elegoo 120pcs Multicolor Dupont Wire 40 pin Male to female, 40 pin male to male, 40 pin female to female breadboard Jumper wires Ribbon cables kit for Arduino	1	$8.86 USD	$8.86 USD	Amazon	Non-specific
Electronic controller	Arduino Uno R3 Microcontroller A000066	1	$18.82 USD	$18.82 USD	Amazon	Non-specific
Extrusion Bath	PTFE-Coated Stainless Steel Dispensing Needles with Luer Lock Connection	5	$2.37 USD	$11.93 USD	McMaster Carr (75165A136)	Non-specific
Extrusion Bath	Thermo Scientific™ Nalgene™ Polypropylene Griffin Low-Form Plastic Beakers	12	$6.78	$81.30 USD	Fisher Scientific Nalgene (02-591-10B)	Non-specific

## 5 Build instructions

After engineering drawings of the device, work was set with the standard parts to assemble the USMA Fiber Device.

To assemble the mechanical components, aluminium sheets were used as support backing to hold the motorized spooling mechanism and maintain vertical alignment with the syringe pump extruder. The back plate was cut into a 65 cm × 20 cm sheet and the base plate was cut into a 42 × 42 cm square. This offered enough support and mass to maintain stability to prevent vibration during fiber spinning. The back plate was bolted into place with two 45 degree aluminium arms adding stability and control (Fig. 1).

The vertical assembly was first assembled (Fig. 2a). The 3 ft stainless steel rod was cut into two pieces each 35 cm long and threaded through two sets of base mount supports. The rods were separated by ~10 cm. The bars were anchored in place by the stainless steel strips cut into rectangles where the ends of the bars were screwed into place. The rods had holes drilled into the center and tapped to ensure the necessary screw threads functioned properly. The two sets of base-mounted shaft supports were used to guide the cut stainless steel rods; this allowed control over height and direction of our spindle device by offering drying time control and also allowed space to insert other baths or textile mechanisms to optimize fiber characteristics. The shaft collars were used as a safety stop and as a movable collar to lock the spindle system along the rods to control the height of drawing.

The spooling system was made by anchoring two base mounted shaft supports to the SureMotion linear actuator assembly spaced at the distance between the stainless steel rods. This allowed for the linear actuator assembly to slide along the rods to change the height range of the fiber drying. The linear actuator was driven by a SureStep stepper motor to drive the screw to move the second SureStep stepper motor that drives the spooling mechanism. The second SureStep stepper motor was attached to a plate that held the motor in place (Fig. 2a). The 566 Carbon Steel Linear Motion Shaft was cut and used as the dowel that attached the 3D printed spool (Fig. 2a i-ii).

The spool was designed using CAD software and 3D printed from polylactic acid (PLA) (https://doi.org/10.17605/OSF.IO/RKY2S). The system was designed to allow for access to five different spools while operating, with a specific number of rotations of fiber to be wrapped around each spool. This was completed by adding valleys into the spool perpendicular to the axis of rotation allowing individual fibers to lay within the valley. Additionally, for applications in our lab, bundles of controlled length were required and we introduced horizontal valleys that are parallel to the axis of spool rotation allowing for easy knot tying / suturing and fiber grouping (Fig. 2a i-ii).

Control of fiber extrusion was completed by anchoring a 25 gauge Teflon coated needle (McMaster-Carr) that is epoxied to a hole in the center of a 150 mL plastic Nalgene Polypropylene beaker (Fig. 2b) that rests in a ring to support the liquid bath and control the height and direction of the fiber bundling (Fig. 2c-d). The cup assembly was supported by an aluminium ring (Fig. 2b-d).

The USMA Fiber Device is mounted on a lab bench or within a temperature- and humidity-controlled chamber for further use (Fig. 3). The temperature was controlled between 20 and 30  °C and a humidity range between 30 and 70%. Too humid conditions did not allow the fibers to dry after extrustion through the precipitation bath, and humidity that was too dry caused the fibers to be brittle and break after extrusion.

The software to control the spooling motors and motorized linear actuator was controlled by an Arduino UNO (Fig. 3b-c). The system had a feedback loop was housed in an OKW electronic enclosure box. The box contained the Sunfounder serial shields, ZJchao 9 V power adaptor, and two control screens (Fig. 3c). The system had dual power switches to control power to the motors using a SureStep power supply, microstepper driver, and power to the Arduino. The system allowed for resetting capabilities with a single toggle switch, and uniform control over the rotation rate of the stepper motor to control drawing rate. Changes to the system were visualized on Sunfounder serial shields LCD screens found in the front of the enclosure box. Exiting from the controller box were 40-pin Elegeo cables and SureStep extension cables that connected to the stepper motors. The schematic diagram of the electronic setup is shown in Fig. 4.

## 6 Operation instructions

The USMA Fiber Device is composed of two major components: the extrusion system, and the mechanical spooling mechanism (Fig. 3). For optimal success of fiber drawing, the system should be housed in a temperature- and humidity-controlled chamber as, especially for biomaterials, ambient environments may affect drying which will alter the fiber diameter and composition.

The required program should be loaded onto the Arduino UNO with the required number of turns per spool (https://doi.org/10.17605/OSF.IO/RKY2S). To ensure safe use (as wet chemicals will be close by), make sure the electronic housing is in a dry and stable lab bench. The main power supply can be switched on and the two front toggle switches can be set to the ON position. The required spool rate is set beforehand by using the “Up” and “Down” buttons on the electronic control box as these cannot be altered during wet-spinning. To active the device, press the “Continue” button. An LED will flash until the Arduino UNO is ready to run.

For the application of drawing collagen fibers, a collagen solution was made from fibrillary collagen (Collagen Solutions, Glasgow Scotland, UK) diluted with acetic acid to concentrations ranging 10 to 20 mg/ml. A 1 to 5 mL solution was loaded into a syringe on the syringe pump. In our experiments, the extrusion bath / cup held a mixture of acetone and ammonium hydroxide (50:1) allowing for precipitation and crosslinking of collagen upon extrusion. The collagen solution was pumped through the 25 gauge Teflon coated needle (McMaster-Carr). Fiber was collected with tweezers at the top of the needle and drawn by hand until the length of the fiber touched the spool. The fiber was then connected to the spool and the “Continue” button is pressed on the electronic system. The system runs until the fiber spooling is completed.

## 7 Validation and characterization

Proper use of the equipment was determined by the number of fibers spooled each time and the measured diameter of each fiber by scanning electron microscope (Hitachi TM-3000 Scanning Electron Microscope, Tokyo, Japan). The consistency of the fibers derived from different concentrations was also assessed using ImageJ (NIH) software. Preliminary studies were conducted using low concentration collagen and a modified device which produced aligned fibers (Fig. S1). Preliminary studies used a similar device setup with different spooling mechanism and spooling rate. The device was capable of generating aligned fibers of controllable diameter (Fig. S2). Furthermore, in order to better understand the air drying of the acetone-precipitated fiber, supercritically drying of the fibers was conducted on the wet fiber. The initial coagulation generates a hydrogel with the collagen solution creating a network of nanofibrils that collapse during the evaporative drying of the acetone/ ammonium hydroxide solution forming the solid collagen fibers produced by this device (Fig. S3). Subsequent studies were conducted using fibrillar collagen (65 mg/ml) (Collagen Solutions, Glasgow Scotland, UK) diluted to 20 mg/ml, 15 mg/ml, and 10 mg/ml with 2% acetic acid and extruded through the USMA Fiber Device. The volumetric flow rate and spooling rate were maintained constant at each concentration. The fiber diameter and length produced from this device was optimally 30 µm and 3 m respectively (Fig. 5). Here we were able to make consistent fibers with tuneable bundles of any number for fibers ranging from a single rotation to 40 rotations based on a single code modification found in the programming of the Arduino software.

## 8 Discussion

Controlling length and diameter of biomaterial fibers produced from a wet-spinning apparatus require control over drawing rate, direction, and alignment which allows for more uniform and consistent fibers to improve tissue engineering projects where precise biomaterial scaffolds are required. Here we have engineered a laboratory system worth ~$2000 with the necessary requirements compared to industrial and scale-up wet-spinning fiber systems. This device allows for control of diameter, spool rate, spool diameter, material, and extrusion/crosslinking bath that are required for versatile research. Biomaterial and synthetic fiber formation is a growing field, especially, when focused on personalizing chemical surfaces and crosslinking capabilities. This system allows the user to experiment with other variables such as hydrophobicity and molecular length not discussed in this paper where uniform fiber length and surface chemistry are required. The capabilities of a device with the Arduino UNO software allow this system to be easily interchangeable and modified by the user making this system useful for several different research projects in a single laboratory.

## Declaration of Competing Interest

The authors declare that they have no known competing financial interests or personal relationships that could have appeared to influence the work reported in this paper.

## References

[1] Lee K.Y., Mooney D.J. (2001). Hydrogels for tissue engineering. Chem. Rev..

[2] Pachence J.M. (1996). Collagen-based devices for soft tissue repair. J. Biomed. Mater. Res..

[3] Zhu J., Marchant R.E. (2011). Design properties of hydrogel tissue-engineering scaffolds. Expert Rev. Med. Devices.

[4] Drury J.L., Mooney D.J. (2003). Hydrogels for tissue engineering: scaffold design variables and applications. Biomaterials.

[5] Tan J., Saltzman W.M. (2004). Biomaterials with hierarchically defined micro- and nanoscale structure. Biomaterials.

[6] Bognitzki M., Czado W., Frese T., Schaper A., Hellwig M., Steinhart M., Greiner A., Wendorff J.H. (2001). Nanostructured fibers via electrospinning. Adv. Mater..

[7] Agarwal S., Wendorff J.H., Greiner A. (2008). Use of electrospinning technique for biomedical applications. Polymer.

[8] Matthews J.A., Wnek G.E., Simpson D.G., Bowlin G.L. (2002). Electrospinning of collagen nanofibers. Biomacromolecules.

[9] Wang C.-Y., Zhang K.-H., Fan C.-Y., Mo X.-M., Ruan H.-J., Li F.-F. (2011). Aligned natural-synthetic polyblend nanofibers for peripheral nerve regeneration. Acta Biomater..

[10] Meyer M., Baltzer H., Schwikal K. (2010). Collagen fibres by thermoplastic and wet spinning. Mater. Sci. Eng. C.

[11] Arafat M.T., Tronci G., Yin J., Wood D.J., Russell S.J. (2015). Biomimetic wet-stable fibres via wet spinning and diacid-based crosslinking of collagen triple helices. Polym. (United Kingdom).

[12] Yaari A., Schilt Y., Tamburu C., Raviv U., Shoseyov O. (2016). Wet spinning and drawing of human recombinant collagen. ACS Biomater. Sci. Eng..

[13] Sionkowska A., Skrzyński S., Śmiechowski K., Kołodziejczak A. (2016). The review of versatile application of collagen. Polym. Adv. Technol..

[14] Parenteau-Bareil R., Gauvin R., Berthod F. (2010). Collagen-based biomaterials for tissue engineering applications. Materials (Basel).

[15] Shoulders M.D., Raines R.T. (2009). Collagen structure and stability. Annu. Rev. Biochem..

[16] DeRosa K.E., Siriwardane M.L., Pfister B.J. (2011).

