# Supplementary material for: Uniform wet-Spinning Mechanically Automated (USMA) fiber device
Source: HardwareX. 2020 Jul 23;8:e00124. doi: 10.1016/j.ohx.2020.e00124 (PMC9041263; doi:10.1016/j.ohx.2020.e00124)
Supplement: Supplementary data 1 [file mmc1.docx]

**Supporting Information**

**Title:** *Uniform wet-Spinning Mechanically Automated (USMA) Fiber Device*

**Authors: Alexander N. Mitropoulos^1, 2^, Kylor Kiesewetter^2^, Eric Horne^3^, Jeff Butler^3^, Joseph R. Loverde^2^, J. Kenneth Wickiser^2,4^**

**Affiliations:**

**^1^ Department of Mathematical Sciences, United States Military Academy, West Point, NY 10996**

**^2^ Department of Chemistry and Life Science, United States Military Academy, West Point, NY 10996**

**^3^ Department of Civil and Mechanical Engineering, United States Military Academy, West Point, NY 10996**

**^4^ Academic Research Division, United States Military Academy, West Point, NY 10996**

**Contact email:** [*alexander.mitropoulos@gmail.com*](mailto:alexander.mitropoulos@gmail.com)*,* [*ken.wickiser@westpoint.edu*](mailto:ken.wickiser@westpoint.edu)

Control over fiber shape and diameter was conducted on several iterations by controlling the spooling rate, concentration of collage, temperature of drying, and the humidity during drying. After optimization, the collagen fibers produced cylindrical fibers that were aligned in bundles with consistent diameters and lengths.

**Figure S1.** Fiber formation after drying. Scanning electron microscope images of collagen fibers at a) 100x, b) 500x, c) 1000x, and d) 5000x magnification.

Initial spooling studies were conducted to produce aligned fibers on a 3D printed spool. Fiber diameter could be controlled by changing the concentration of the collagen solution. Fiber alignment and shape were critical to develop a successful spooling device.

**Figure S2.** Evaluation of collagen concentration and fiber spooling. a) Images of (i) spooled collagen fibers and (ii) aligned fibers (iii-iv) under scanning electron microscopy. b) Scanning electron microscopy images of collagen fibers at (i) 6 mg/ml and (ii) 7.5 mg.ml concentrations. c) Plot of average fiber diameter versus collagen thickness.

Air drying after exposure to the bath causes the fibers to dry and the porous network of the fiber to collapse generating cylindrical fibers. Studies were conducted on the surface of the collagen fiber before air drying to determine how the cylindrical structure formed. Fibers were coagulated in the acetone/ammonium hydroxide bath and not allowed to air dry. To keep the porous structure, the fibers were dehydrated in ethanol and supercritically dried in CO_2._ Before air drying, the collagen fibers are a network of nanofibrils that collapse due to capillary forces. The loss of the aqueous solution causes approximately a 10-fold reduction in fiber diameter.

**Figure S3.** Scanning electron microscope image of a) supercritically dried collage fiber after coagulation but before drying. (b-d) The collagen fibers are a porous network of collagen fibrils caused by the aggregation of the acetone and ammonium hydroxide bath.
